# In Vivo Anti-Inflammatory Activity of Four Edible Cactaceae Flowers from Mexico

**DOI:** 10.3390/metabo15100665

**Published:** 2025-10-11

**Authors:** Christian Alfredo Pensamiento-Niño, Alma Delia Hernández-Fuentes, Javier Añorve-Morga, Arturo Duarte-Sierra, Esther Ramírez-Moreno, Carolina Guadalupe Sosa-Gutiérrez, Deyanira Ojeda-Ramírez

**Affiliations:** 1 Área Académica de Ciencias Agrícolas y Forestales, Instituto de Ciencias Agropecuarias, Universidad Autónoma del Estado de Hidalgo, Avenida Universidad 133, San Miguel Huatengo, Santiago Tulantepec de Lugo Guerrero 43775, Mexico; christian_pensamiento@uaeh.edu.mx; 2 Área Académica de Ingeniería en Alimentos e Ingeniería Agroindustrial, Instituto de Ciencias Agropecuarias, Universidad Autónoma del Estado de Hidalgo, Avenida Universidad 133, San Miguel Huatengo, Santiago Tulantepec de Lugo Guerrero 43775, Mexico; 3 Área Académica de Química, Instituto de Ciencias Básicas e Ingeniería, Universidad Autónoma del Estado de Hidalgo, Mineral de la Reforma 42184, Mexico; 4 Department of Food Science and Horticultural Research Centre, Laval University, Québec, QC G1V 0A6, Canada; 5 Área Académica de Nutrición, Instituto de Ciencias de la Salud, Universidad Autónoma del Estado de Hidalgo, Circuito Actopan Tilcuautla s/n, Ex hacienda La Concepción, San Agustin Tlaxiaca, Pachuca 42160, Mexico; 6 Área Académica de Medicina Veterinaria y Zootecnia, Instituto de Ciencias Agropecuarias, Universidad Autónoma del Estado de Hidalgo, Avenida Universidad 133, San Miguel Huatengo, Santiago Tulantepec de Lugo Guerrero 43775, Mexico

**Keywords:** anti-inflammatory activity, biological activity, edible flowers, hydroethanolic extract, 12-*O*-tetradecanoylphorbol-13-acetate, phenolic compounds

## Abstract

Background/Objectives: The therapeutic properties of edible flowers are widely used to improve human health. The phenolic compounds present in edible flowers, such as phenols and flavonoids, among others, play an important role as effective antioxidant compounds against diseases related to oxidative stress. These compounds exhibit biological activities such as anti-ulcerogenic, antimicrobial, neuroprotective, anti-cancer, and anti-inflammatory properties. The objective of this study was to evaluate the in vivo anti-inflammatory activity of hydroethanolic extracts of four Mexican cacti flowers. Methods: A hydroethanolic extract was obtained via maceration for each cactus flower and evaluated using a model of edema induced in mouse ears by 12-*O*-tetradecanoylphorbol-13-acetate (TPA) as a guide for the anti-inflammatory activity. Compounds in cacti flower extracts were quantified by HPLC. Results: All of the hydroalcoholic flower extracts showed an anti-inflammatory effect. The greatest effect of inhibition of auricular edema (61.2 ± 4.23%) was observed in the group of mice treated with the Cardon extract at a dose of 3 mg/ear. This effect can be attributed to the main compounds detected by HPLC in the extract such as *p*-coumaric acid, catechin, kaempferol, and quercetin. These compounds are involved in the inhibition of pro-inflammatory mediators and enzymes such as cyclooxygenases and lipoxygenases. Conclusions: This preliminary evidence supports further preclinical evaluation of the Cardon flower.

## 1. Introduction

Inflammation is a biological process that is activated after an alteration of tissue structures. It is a physiological response of protection of the organism to a physical, chemical, or biological stimulus. The process is triggered by tissue lesions and infections, microbial invasions, and some antigens to initiate homeostatic regulation that involves various mediators (cells and extracellular molecules) of great importance for the survival of the human being. The main objective of inflammation is to eliminate the damaging agent and start the tissue restoration process [1].

In addition, inflammatory diseases, such as gout, atherosclerosis, respiratory, and cardiovascular conditions, are related to the prolonged production of reactive oxygen species (ROS). These metabolites (reduced oxygen) are dangerous to cells at high concentrations, causing tissue damage, in addition to being able to act as signaling molecules and inflammatory mediators [2].

On the other hand, anti-inflammatory drugs of synthetic origin have certain limitations related to cost and serious side effects. Due to the great chemical diversity present in plants, they have become viable alternative sources to obtain compounds with anti-inflammatory effects. For hundreds of years, natural products have played a fundamental role in the therapeutic treatment of diseases [3].

The health benefit granted by the use and consumption of edible and medicinal flowers is attributed to the presence of antioxidant compounds that can exert a preventive effect against diseases associated with oxidative stress [4]. Such effects depend on the concentration of aromatic and/or polyhydroxylated compounds such as phenols, flavonoids, and alkaloids [5]. According to the literature, the main natural compounds present in flowers with an antioxidant effect are phenolic acids, flavonoids, and their derivatives, such as delphinidin, delphinidin 3-*O*-sambubioside, rutin, lutein, and quercetin 3-*O*-rhamnoside [6].

Cactaceae is a family of xerophytes that inhabit arid lands of the Americas, especially the arid and semiarid environments of Mexico, and to a lesser extent and in Brazil, Argentina, and Chile [7,8]. Mexico has 52 genera and 850 species of cactaceae, 84% of them are endemic [7]. Cacti fruits and other edible parts are a good source of protein, carbohydrates (arabinose, rhamnose, galactose, xylose, mannose, glucose, and galacturonic acid), lipids (myristic, stearic, oleic, palmitic, linoleic, and linolenic acids), fiber, vitamins (C, E, B1, B2, B3, B6, B9, and B12), and minerals (Ca, Mg, K, P, Fe, Cu, Se, and Mn) [9,10]. Furthermore, they contain bioactive compounds as betalains, phenolic compounds, carotenoids, phytosterols, tocopherols, and terpenes [7,9].

Most studies about the nutritional and pharmacological potential of cacti focus only on their fruits and stems. Recently, our working group reported the nutritional and antioxidant potential of four cacti flowers [11]; however, it is necessary to carry out more studies on the possible therapeutic uses of these and identify the active principles. Due study oxidative stress being closely related to the inflammatory process, the aim of this research was to evaluate the in vivo anti-inflammatory effect of hydroethanolic extracts of four cacti flowers using a model of mice edema induced by 12-*O*-tetradecanoylphorbol-13-acetate (TPA) to assess its possible use as an adjuvant in the inflammatory process.

## 2. Materials and Methods

### 2.1. Plant Material

The flowers of Cardon (*Cylindropuntia rosea*) (CA), Xoconostle, ulapa variety (*Opuntia oligacantha*) (XU), Xoconostle, cuaresmeño variety (*Opuntia matudae*) (XC), and Pitaya (*Echinocereus cinerascens*) (PI) were obtained in the municipality of Tetepango, Hidalgo, Mexico, located at 20°06.63′ N; 99°09.18′ W at an altitude of 2100 m above sea level, on June 2021 during flowering. The plants were authenticated by Biologist Oscar Castelán, Area Academica de Ciencias Agricolas y Forestales, Universidad Autonoma del Estado de Hidalgo, Mexico. A voucher specimen was deposited in the Area Academica de Ciencias Agricolas y Forestales, Universidad Autonoma del Estado de Hidalgo, Mexico (code numbers: 0100 ACAF, 0101 ACAF,010 ACAF and 0103 ACAF). They were stored in a deep freezer at −76 °C (Thermo-Scientific, 703, Waltham, MA, USA), subsequently freeze-dried (Labconco, 7948000, Kansas City, MO, USA), pulverized in a blade mill (Knife Mill Grindomix GM 200; Hahn, Germany), and stored refrigerated (4 °C) until analysis.

### 2.2. Preparation of Hydroalcoholic Extracts

A total of 15 g of dried flowers was extracted via maceration with 250 mL of an ethanol/water mixture (70:30, *v*/*v*) for one week. Next, the extracts were filtered through filter paper (Whatman, 11 μm). Then, the solvent was evaporated at reduced pressure in a rotaevaporator (BÜCHI, R-215, Flawil, Switzerland). Finally, the dry hydroalcoholic extract of Cardon (CAHE), the hydroalcoholic extract of Xoconostle ulapa, (XUHE), the hydroalcoholic extract of Xoconostle cuaresmeño (XCHE), and the hydroalcoholic extract of Pitaya (PIHE) were stored at 4 °C in amber vials for two weeks until biological testing with the following batch codes: C211109 for CAHE, U211116 for XUHE, C211123 for XCHE, and P211130 for PIHE.

### 2.3. Quantification of Phenolic Compounds by HPLC

Phenolic acids and flavonoids were quantified by high-performance liquid chromatography (HPLC) using an Agilent 1100 HPLC equipment, coupled to a diode array detector (Model 1100 series, Agilent Technologies, Santa Clara, CA, USA), according to Joaquín-Ramos et al. [12].

For the identification and quantification of phenolic acids, samples (50 mg of extract/mL ethanol) were injected into a Nucleosil 100 SA column (125 × 4.0 mm i.d., 5 µm particle size, Macherey-Nagel, Düren, Germany). The mobile phase consisted of H_2_O at pH 2.5 with trifluoroacetic acid (TFA) as solvent A and acetonitrile as solvent B. The elution system was a gradient as described below: 85% A, 10 min; 65% A, 20 min; 65% A, 23 min. The flow was maintained at 1 mL/min at 22 °C and the sample injection volume was 20 µL. The detection of compounds was performed at λ 254, 280, and 330 nm. The standards used were *p*-hydroxybenzoic, chlorogenic, ferulic, gallic, *p*-coumaric, protocatechuic, sinapic, rosmarinic, *β*-resorcylic, and vanillic acids (Sigma Aldrich, St. Louis, MO, USA). Calibration curves were made with the standards in a concentration range of 0.001–25 µg/mL. All the calibration curves were linear (R^2^ = 0.981–0.998).

On the other hand, for the identification of flavonoids, an Agilent Hypersil ODS column (125 × 4.0 mm i.d., 5 μm particle size, Wilmington, DE, USA) was used. The used eluents were those mentioned above, and the following elution gradient was used: 65% A, 10 min; 65% A, 20 min; 65% A, 25 min; and a flow temperature at 25 °C. Apigenin, catechin, phloretin, phloridzin, isorhamnetin, kaempferol, myricetin, naringenin, and quercetin were used as standards (Sigma Aldrich, St. Louis, MO, USA).

For the quantification of each of the phenolic acids and flavonoids, calibration curves were made with the standards in a concentration range of 0.001–25 µg/mL. The results were expressed as μg/g of dry extract. All the calibration curves were linear (R^2^ = 0.985–0.998) within the ranges of the concentrate selected.

The chromatographic methods were validated according to ICH guideline Q2 (R1). The linearity of methods was demonstrated by the coefficient correlation of standard curves. The precision and accuracy were established by evaluations, recoveries, and standard deviation of three concentrations (0.375, 0.250, and 0.125 µg/g of standard). The limit of detection (LOD) and quantitation (LOQ) were calculated as 3 times the standard deviation at the lowest centration. The quantification limit was 10 times the standard deviation from the stock used at the lowest concentration.

### 2.4. In Vivo Anti-Inflammatory Activity

In vivo anti-inflammatory activity was determined according to Rivero-Pérez et al. [13] with slight modifications.

Twenty-four male CD-1 strain mice, with a weight between 25 and 30 g, were used. The mice were maintained under standard conditions (12 h light/dark cycle at 22 °C and 45% relative humidity), following the Mexican Official Standard NOM-062-ZOO-1999: Technical specifications for the production, care, and use of laboratory animals [14], in addition to being approved by the ethics committee for the care and use of laboratory animals of the Autonomous University of the State of Hidalgo (approval number: CICUAL/006/2021). The minimum number of animals and the duration of observation required to obtain consistent data were used.

Mice were randomized into 6 groups using a simple method, with 4 mice in each. Firstly, mice were intraperitoneally anesthetized with Zoletil^®^ (Tiletamine/Zolazepam) at a dose of 100 mg/kg. Subsequently, 20 µL of 12-*O*-tetradecanoylphorbol-13-acetate (TPA) (2.5 µg dissolved in 20 µL of ethanol) was applied on the inner and outer surface of the left ear (10 µL/side). Then, the treatments were applied with the same procedure described above (10 µL/side). The hydroalcoholic extracts of Cardon (CAHE), Xoconostle ulapa (XUHE), and Xoconostle cuaresmeño (XCHE) were administered at doses of 3 mg/20 μL of ethanol; the Pitaya extract (PIHE) was administered at 2 mg/20 μL ethanol due solubility problems. Indomethacin (1 mg/20 µL acetone/ear) was used as a positive control, and the negative control group did not receive treatment. In the right ear of the mice, only the respective vehicle was applied, as shown in Table 1.

Six hours after application of the irritant agent (TPA), the animals were sacrificed via cervical dislocation. Subsequently, a circular segment approximately 6 mm in diameter was cut from the treated (t) and untreated (nt) ears and weighed. Finally, the % inhibition of inflammation exerted by the extracts was determined using the following equation:

% inhibition = [(Δw_control − Δw_treated)/Δw_control] × 100

where Δw = wt − wnt, with wt being the weight of the treated ear and wnt being the weight of the untreated ear.

To minimize variation due to technique, a single investigator performed the measurements throughout any one experiment. A blinded outcome assessment was used to avoid any bias.

### 2.5. Statistical Analysis

The data were normalized in log(10) for the statistical analysis. The statistical significance (*p* < 0.05) was determined for the pharmacological data using ANOVA with the Tukey multiple comparisons test. Statistical analysis was performed using JMP software 5.0.1 (SAS Institute Inc., Cary, NC, USA). 

## 3. Results

The ground CAHE, XUHE, XCHE, and PIHE resulted in 79, 132, 106, and 202 mg/g of dry matter, respectively.

**Figure 1:**
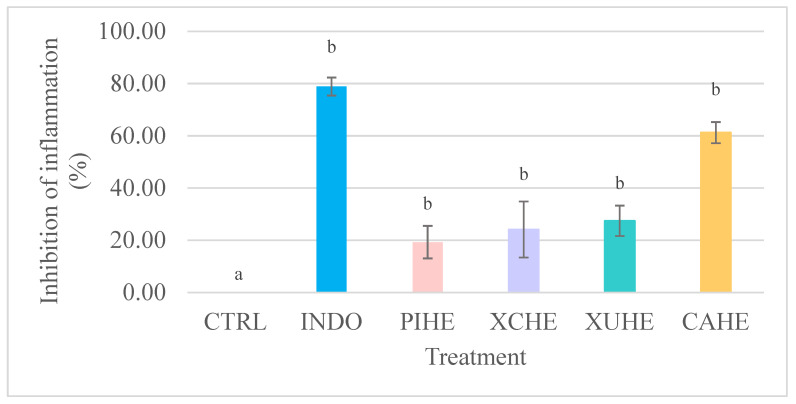
Inhibition of inflammation exerted by hydroalcoholic extracts of Cactaceae flowers on CD-1 mouse ear edema induced by TPA. CAHE: Cardon (*Cylindropuntia rosea*) hydroalcoholic extract; XUHE: Xoconostle ulapa (*Opuntia oligacantha*) hydroalcoholic extract; XCHE: Xoconostle cuaresmeño (*Opuntia matudae*) hydroalcoholic extract; PIHE: Pitaya (*Echinocereus cinerascens*) hydroalcoholic extract; Indo: indomethacin (positive control); Ctrl: negative control group. The values represent the mean ± standard deviation (*n* = 4). Different lowercase letters in the bars indicate statistically significant differences (*p* ≤ 0.05) with the negative control.

Cactus flower extracts were evaluated to determine their anti-inflammatory effect in vivo using a model of edema induced by TPA in CD-1 mouse ears (Figure 1). All the treatments showed significant effects (*p* ≤ 0.05) on the inhibition of auricular edema with respect to the negative control.

The highest anti-inflammatory effect (61.2 ± 4.23% II) was observed for the hydroalcoholic extract of Cardon (*Cylindropuntia rosea*), followed by the hydroethanolic extract of Xoconostle ulapa (*Opuntia oligacantha*) and Xoconostle cuaresmeño (*Opuntia matudae*) (27.44 ± 5.83 and 24.13 ± 10.73% II, respectively). Furthermore, CAHE did not show any statistically significant difference with respect to the positive control indomethacin (see Supplementary Data Stastistical_analysisS1). On the other hand, the hydroethanolic extract of Pitaya (*Echinocereus cinerascens*) presented 19.29 ± 6.22% inhibition of auricular edema; however, it should be noted that a lower dose (2 mg/ear) was used.

**Figure 2:**
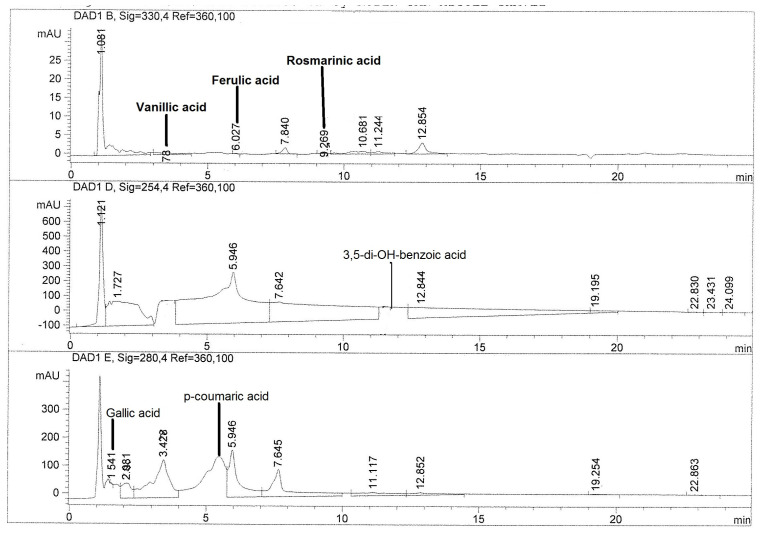
HPLC chromatograms of phenolic acid profile of hydroalcoholic extract of Cardon (*Cylindropuntia rosea*) (CAHE).

**Figure 3:**
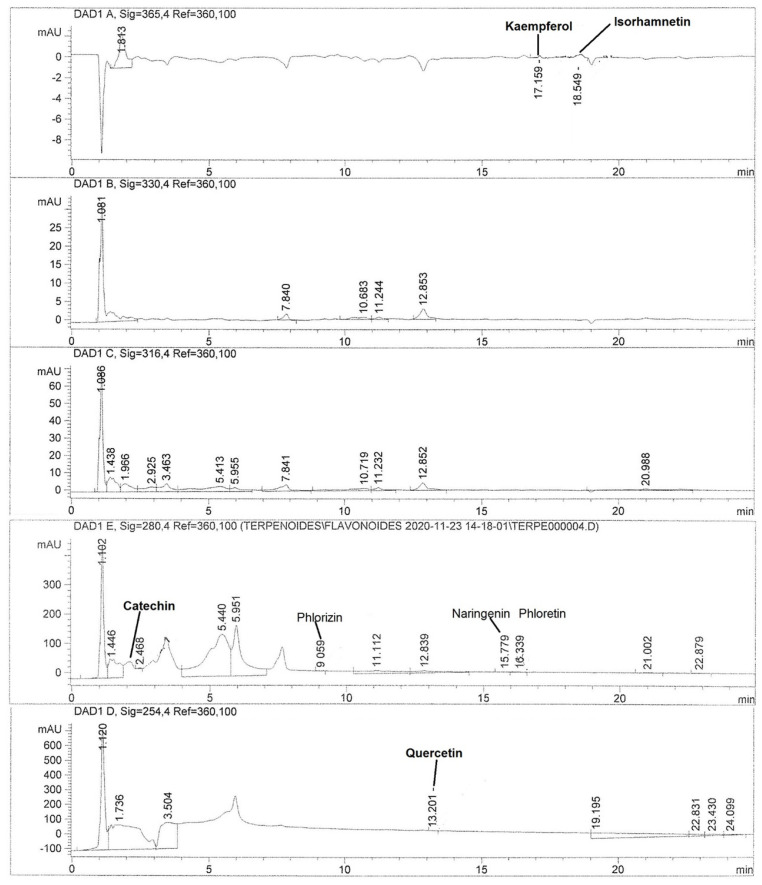
HPLC chromatograms of flavonoid profile of hydroalcoholic extract of Cardon (*Cylindropuntia rosea*) (CAHE).

To investigate the possible metabolites responsible for the anti-inflammatory effect of cacti flowers, quantification of phenolic acids and flavonoids in the extracts was performed. Figure 2 and Figure 3 show chromatograms of the most active extract, CAHE.

Table 2 shows the quantification by HPLC of phenolic compounds present in the hydroalcoholic extract of four cacti flowers. Cardon (*Cylindropuntia rosea*) and Xoconostle ulapa (*Opuntia oligacantha*) hydroalcoholic extracts showed the greatest presence of compounds. Regarding phenolic compounds, chlorogenic acid was detected only in the Pitaya (*Echinocereus cinerascens*) hydroalcoholic extract (PIHE), while vanillic acid was only present in the Cardon (*Cylindropuntia rosea*) extract (CAHE). Additionally, CAHE showed the highest content of *p*-coumaric acid, while XUHE had the highest content of ferulic and rosmarinic acids. Finally, the Pitaya (*Echinocereus cinerascens*) hydroalcoholic extract (PIHE) showed the greatest content of 3,5-di-OH-benzoic, gallic, protocatechuic, and *β*-resorcylic acids.

Additionally, CAHE showed the highest quantity of phloretin, isorhamnetin, and naringenin, while catechin, kaempferol, and quercetin were found in PIHE, XUHE and XCHE, respectively. Apigenin and myricetin were detected only in XUHE.

Regarding the extract with the highest anti-inflammatory effect, CAHE, the most abundant phenolic acids were *p*-coumaric acid, gallic acid, and vanillic acid (75.13, 2.85, and 1.90 μg/g of dry extract, respectively). Meanwhile, the flavonoids identified in the highest concentration were quercetin, isorhamnetin, and catechin with values of 1.94, 1.65, and 1.44 μg/g of dry extract, respectively.

## 4. Discussion

Mexico is characterized by a remarkable richness in plants of the *Opuntia* genus, with around 1500 species with a commercial importance, mainly due to their fruits. They have been used since ancient times in folk medicine for the treatment of various diseases. These plants are currently considered a source of nutritional compounds and phytochemicals with beneficial effects on human health [15], and the flowers are of great interest due to the presence of bioactive compounds [16]. Recently, our research group demonstrated the antioxidant capacity of Cardon cacti (*Cylindropuntia rosea*), Xoconostle ulapa (*Opuntia oligacantha*), Xoconostle cuaresmeño (*Opuntia matudae*), and Pitaya (*Echinocereus cinerascens*) flowers [11]. However, the anti-inflammatory potential of these cacti flowers has not been explored until now.

The search for new therapeutic alternatives in advanced medicine leads to experimenting with appropriate and effective models to find the best application of the metabolites of interest. The main in vivo models currently used in the evaluation of the anti-inflammatory activity (acute phase) of plant extracts are as follows: plantar edema induced by carrageenan, bradykinin, dextran, histamine/5-HT, and lipopolysaccharide (LPS); ear edema induced by oxazolone, croton oil, and 12-*O*-tetradecanoylphorbol-13 -acetate (TPA); acetic acid/compound 48/80-induced vascular permeability; and the pleurisy model [17]. According to the literature, the main models used to evaluate the anti-inflammatory activity of edible flower extracts are through evaluations in cell cultures (activation of Raw 264.7 macrophages induced by LPS) and in vivo models (mouse ear edema induced by oil Croton, dimethylbenzene, and TPA; carrageenan-induced mouse paw edema and granuloma induced by cotton pellets) [18,19].

The application of TPA in the mouse ear has an inflammatory effect, causing the formation of edema and the activation of neutrophils, defense cells against tissue damage. This irritant agent is used in animal models to evaluate bioactive compounds (natural and synthetic) with anti-inflammatory effects in the acute phase. The mechanism by which it generates inflammation is through the activation of protein kinase C, which triggers the activation of phospholipase A2, prostaglandins, and leukotrienes [17,20].

The findings in our experiment showed that the hydroalcoholic extracts of cacti flowers have anti-inflammatory activity, and the hydroalcoholic extract of Cardon was the most active compared to the rest of the extracts. The Pitaya flower hydroalcoholic extract was the least active. It is important to mention that in this study, no dose–response/ED_50_ was estimated for the extracts, but this will follow in future research.

There are few studies about the anti-inflammatory activity of edible flower extracts tested in topical models induced by TPA. However, Lee et al. [21] evaluated the anti-inflammatory activity of the hydroethanolic extract of the Chrysanthemum flower (*Chrysanthemum indicum* L.) on mouse ear edema. The chrysanthemum extract was obtained by using the reflux method with 70% ethanol; it presented an anti-inflammatory effect by reducing the thickness and weight of the ear produced by TPA, and this effect was comparable to the effect exerted by indomethacin. The anti-inflammatory activity was attributed to the presence of flavones (acacetin, apigenin, and derivatives) in the chrysanthemum flower, which acts by inhibiting pro-inflammatory mediators such as interleukin 1β (IL-1β) and tumor necrosis factor-α (TNF-α); in the prolonged inflammation phase, these compounds showed 76% inhibition of inflammation at doses of 5 mg/kg. An inhibitory effect was also found in this study for the extracts of Cardon, Xoconostle ulapa, Xoconostle cuaresmeño, and Pitaya, which reduced inflammation considerably in the acute phase.

In addition, Yasukawa et al. [22] evaluated the inhibitory effect of triterpene acetates obtained from the active fraction of the methanolic extract of artichoke (*Cynara cardunculus*) flowers on the inflammation induced by TPA in mouse ears. The method of obtaining the artichoke extract was maceration; subsequently, fractions were made where four triterpenes (α and β-amyrin, taraxasterol, and ψ-taraxasterol) and their corresponding acetates were identified. The dose used to evaluate the anti-inflammatory effect was from 0.02 to 1 mg of extract. With the above, 50% inhibition of auricular edema was obtained by the action of α-amyrin at a concentration of 0.50 μmol/ear (0.213 mg/ear). This effect was smaller than the effect found in this study for the extract of Cardon (*C. rosea*) (CAHE), which inhibited 61.20 ± 4.23% of the inflammation induced by TPA at a dose of 3 mg/ear; however, it is important to mention that in our research, the full extract was applied.

In another study, Li et al. [23] evaluated the anti-inflammatory effect of an ethanolic extract (95% *v*/*v*) of the Jasmine flower (*Jasminum grandiflorum* L.) on TPA-induced ear edema in mice, which inhibited 28.93% of the inflammation at a dose of 2 mg/ear. This effect was similar to that found for the Xoconostle ulapa (XUHE) and Xoconostle cuaresmeño (XCHE) extracts, which decreased inflammation by 27.44 and 24.13%, respectively, at a dose of 3 mg of extract/ear. Regarding the Pitaya flower hydroalcoholic extract (PIHE), a dose of 2 mg of extract/ear was used and it inhibited 19.29% of the inflammation induced by TPA, an effect slightly closer to what was reported for the extract of *J. grandiflorum.*

On the other hand, the anti-inflammatory capacity of some flowers of related species has been evaluated in other animal models. For instance, a methanolic extract of *Opuntia ficus-indica* flowers at a dose of 400 mg/kg decreased paw edema formation and the number of immune cells induced by Carrageenan in Wistar rats; the effect was attributed to phenolic compounds. Furthermore, an extract of stems, flowers, and fruits of *Opuntia dilleniid* at 50 mg/kg inhibited paw edema formation in the same model. This effect was due to the active principles kaempferol-3-*O*-α-arabinoside, isorhamnenin-3-*O*-β-D-glucopyranoside, and isorhamnenin-3-*O*-β-D-rutinose [24]. These reports support our findings; however, it is not possible to make a direct comparison if the anti-inflammatory effects for the animal models are different.

Phenolic acids are biologically active compounds found in different parts of fruits, vegetables, and cereals, and they are also present in drinks and juices. These compounds have the function of protecting the plant against external damage and diseases; however, they are important in health maintenance due to their pharmacological properties, such as having antioxidant, antimicrobial, anticancer, antidiabetic, and anti-inflammatory activity [25]. Another group with important pharmacological activities is flavonoids; these have pharmacological effects such as being antioxidant, antimicrobial, antiproliferative, anticancer, neuroprotective, antiangiogenic, and anti-inflammatory. Regarding the last one property, flavonoids have a fundamental role in the control of mediators responsible for the inflammation process through the inhibition of regulatory enzymes and transcription factors [26]; therefore, quantification of phenolic acids and flavonoids was performed by HPLC for all cacti flower extracts (Table 2).

There is scarce information about the identification and quantification of phenolic compounds in cactus flowers. In fact, only two of the phenolic acids (ferulic and *p*-coumaric acids) and one flavonol (kaempferol) of the list in Table 2 have been reported in flowers of the *Opuntia* genus (*O. ficus-indica*, *O. stricta*, *O. ficus-barbarica*, *O. robusta*, *O. mycrodasys*, *O. engelmannii*, *O. hyptiacantha*, *O. streptacantha*, *O. megacantha*, and *O. albicarpa*). The ferulic acid content has been reported as between 291 and 786 µg/g of extract, *p*-coumaric acid from 65 to 178 µg/g of extract, and kaempferol from 321 to 708 µg/g of extract. The values found in our study for ferulic acid (0.4 µg/g), *p*-coumaric acid (1.6–75.13 µg/g), and kaempferol (1.65–2.61 µg/g) are much less; this could be due to the cactus species, as well as the place and period of collection.

On the other hand, gallic acid, *p*-coumaric acid, and quercetin were detected in all analyzed flowers, while chlorogenic acid only was detected in *E. cinerascens*, vanillic acid in *C. rosea*, and apigenin and myricetin in *O. oligacantha* (Table 2). The major compound in PIHE was protocatechuic acid, followed by 3,5-di-OH-benzoic acid, catechin, gallic acid, and *p*-coumaric acid. Meanwhile, in XUHE, it was rosmarinic acid, followed by gallic acid, kaempferol, *β*-resorcylic acid, and apigenin. In XCHE, the main phenolic compounds were quercetin, protocatechuic acid, gallic acid, *β*-resorcylic acid, and *p*-coumaric acid. Finally, the most abundant compound in the extract with the highest anti-inflammatory effect (CAHE) was *p*-coumaric acid, followed by gallic acid, quercetin, vanillic acid, kaempferol, and catechin.

**Figure 4:**
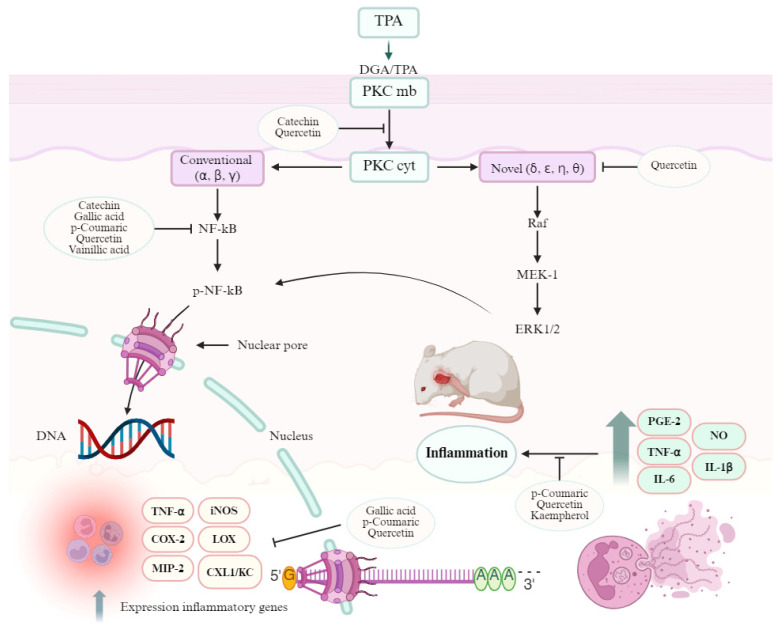
The implication of 12-*O*-tetradecanoylphorbol-13-acetate (TPA) in inflammation. TPA receptor activation induces the translocation of conventional and novel PKCs, which activate the mitogen-activated protein kinases (MAPKs) and nuclear factor-κB (NF-κB) intracellular pathways, producing overexpression of inflammatory genes. Compounds identified in *Cylindropuntia rosea* hydoalcoholic extract (CAHE) can act on several points of the process. Quercetin can inhibit PKCδ translocation, binds to the catalytic domain of PKCs, and inhibit PKθ phosphorylation. Catechin inhibits PKC-dependent NADPH activation. Kaempferol binds to the catalytic domain of PKCα. Quercetin, kaempferol, catechin, and *p*-coumaric, gallic, and vanillic acids can inhibit the activation of NF-κB. Quercetin, *p*-coumaric acid, and gallic acid inhibit inflammatory gene expression. Finally, quercetin, kaempferol, and *p*-coumaric acid can inhibit several inflammatory enzymes.

There are strengthened links between TPA-PKC-COX/LOX pathways. Phorbol esters, including TPA, are analogues of DAG and cause translocation and activation of protein kinase C (PKC) [27]. There are three families of PKCs: conventional PKCs that include PKCα, PKCβ, and PKCγ; novel PKCs that includes PKCδ, PKCε, PKCη, and PKCθ; and atypical PKCs (PCKζ, PCKλ/ι). TPA has an effect on the first two types. Protein kinase C activation is a complicated process that includes membrane association of the enzymes, priming by phosphorylation, conformational changes induced by binding of proteins or second messengers, and the release of a pseudo substrate [28]. The stimulation of PKC isoforms by TPA plays a pivotal role in inflammation as it activates the mitogen-activated protein kinases (MAPKs) and nuclear factor-κB (NF-κB) intracellular pathways, as well as generating mediators such as interleukin (IL)-1β, tumor necrosis factor (TNF)-α, macrophage inflammatory protein (MIP)-2, prostaglandins, and keratinocyte-derived chemokine (CXCL1/KC), among others [29]. The phenolic compounds identified in Cardon (*Cylindropuntia rosea*) flower can inhibit several points of the process [30] and the inflammation caused by TPA (Figure 4).

*p*-Coumaric acid is a phenolic compound that is present in fruits, vegetables, and cereals, in free or conjugated form; antioxidant, antidiabetic, antiviral, antifungal, antimelanogenic, and anti-inflammatory properties are attributed to it [31]. Regarding the anti-inflammatory effect, it has been reported that *p*-coumaric acid (10–40 mg/mL) is able to inhibit the inflammatory mediator’s cytokines such as cyclooxygenase 2 (COX-2), inducible nitric oxide synthase (iNOS), and interleukin 1-β (IL-1β); enzymes such as metalloproteinases (MMP1, MMP3, and MMP13); aggrecanases (ADAMTS4 and ADAMTS5); and reactive oxygen species [32]. In addition, it inhibits the expression of the NF-κB gene, the activity of TNF-α, and the synthesis of prostaglandin E2 (PGE2) [33].

On the other hand, gallic acid is present in nuts, vegetables, and fruits and it has been linked to antioxidant, antimicrobial, antidiabetic, and anti-inflammatory effects [34]. Gallic acid (50–100 mg/kg) can inhibit the activation of NF-κB, which in turn activates inflammatory mediators such as IL-1β, TNF-α, COX-2, and iNOS. In addition, it inhibits the expression of pro-inflammatory mediators such as nitric oxide (NO), PGE2, and interleukin 6 (IL-6); however, it does not exert a significant effect on COX-2 [34,35].

Regarding vanillic acid, the presence of this compound in some plants has been reported to have various pharmacological properties such as sedative, antidepressant, antinociceptive, antihypertensive, antiulcerative, anticancer, hepatoprotective, antifungal, and antioxidant activity. In this sense, Ziadlou et al. [36] evaluated the anti-inflammatory effect of vanillic acid in a microtissue inflammation model of osteoarthritic human chondrocytes on the inhibition of osteoarthritis signaling pathways. The anti-inflammatory mechanism of action found was through the inhibition of the signaling pathways of the nuclear factor of the kappa light chain of activated B cells (NF-κB) as a result of the attenuation of the phosphorylation of the nuclear factor of the gene of light kappa polypeptide in inhibitory alpha B cells (lκBα) at a dose of 1 μM vanillic acid.

Another group of abundant compounds in the Cardon flower is flavonoids. These have pharmacological effects, such as being antioxidant, antimicrobial, antiproliferative, anticancer, neuroprotective, antiangiogenic, and anti-inflammatory. Flavonoids have a fundamental role in the control of mediators responsible for the inflammation process through the inhibition of regulatory enzymes and transcription factors [26]. The main flavonoids present in the hydroalcoholic extract of the Cardon flower were the quercetin and kaempferol flavonols, followed by the catechin flavanol (Table 2).

Quercetin has pharmacological properties, mainly having anticancer, antiobesity, neuroprotective, antiatherosclerotic, and anti-inflammatory activity. Regarding the latter, quercetin can inhibit TNF-α, enzymes (COX, lipoxygenases (LOX), and inflammatory mediators responsible for the initiation of the inflammatory process [37]. Furthermore, the antioxidant effect of quercetin is essential to eliminate reactive oxygen species (ROS), the main factor responsible for oxidative stress and a pivotal player in the inflammatory process [38].

Moreover, kaempferol is a flavonol which has shown an anti-inflammatory effect through several pathways. For instance, it inhibits the release of pro-inflammatory mediators (IL-6, IL-1, IL-18, IL- 1β, and TNF-α), Toll-like receptor 4 (TLR4), and the binding activity of NF-κB to DNA and myeloid differentiation factor 88. Kaempferol is also known to inhibit the NF-κB and TNF-β activity activating protein 1 (AP-1) and enzymes involved in inflammation, such as LOX, COX-2, and iNOS; additionally, it increases the expression of mRNA and proteins of genes regulated by Nrf2 and inhibits hyaluronidase [24,39,40]. Finally, catechins are polyphenols present in vegetables and plants; epicatechins, epicatechin gallate, epigallocatechin, gallocatechin stereoisomer, and gallocatechin stereoisomer belong to this group. These compounds exhibit pharmacological effects such as being antibacterial, antihypertensive, and anti-inflammatory. The mechanisms of action of the anti-inflammatory effect are mainly through the inhibition of IL-8 secretion due to the suppression of NF-κB activity induced by TNF-α [41].

As we can observe, anti-inflammatory activity is strongly correlated with the compounds present in plant extracts, such as phenolic acids and flavonoids [19]. According to this, the anti-inflammatory activity of Cardon flower extract can be attributed to the phenolic acids (gallic and *p*-coumaric acid) and flavonoids (catechin, kaempferol, and quercetin) found in this study, which inhibit inflammatory mediators such as cyclooxygenase, lipoxygenase, and nitric oxide synthase enzymes.

## 5. Conclusions

Our study could be considered the first to document the anti-inflammatory activity of cactus flowers. In this study, we evaluated the anti-inflammatory potential of the hydroalcoholic extract of four cactus flowers (*Cylindropuntia rosea*, *Opuntia oligacantha*, *Opuntia matudae*, *Echinocereus cinerascens*). The highest effect was observed for *Cylindropuntia rosea* and this effect is due to the presence of *p*-coumaric acid, catechin, kaempferol, and quercetin in the extract. These preclinical results support further dose–response and mechanistic studies and broader phytochemical profiling.

## Figures and Tables

**Table 1:** Treatment and vehicles used in in vivo anti-inflammatory model.

Group	Left Ear	Right Ear
Negative control	TPA + ethanol	ethanol
Positive control	TPA + ethanol/Indometacin + acetone	ethanol + acetone
Cardon flower	TPA + ethanol/CAHE + ethanol	ethanol
Xoconostle ulapa flower	TPA + ethanol/XUHE + ethanol	ethanol
Xoconostle cuaresmeño flower	TPA + ethanol/XCHE + ethanol	ethanol
Pitaya flower	TPA + ethanol/PIHE + ethanol	ethanol

**Table 2:** Content of phenolic acids and flavonoids in cactus flowers’ hydroalcoholic extracts (µg/g of dry extract).

Compounds	CAHE	XUHE	XCHE	PIHE
**Phenolic acids**				
3,5-di-OH-benzoic acid	0.96 ± 0.03 ^b^	0.89 ± 0.00 ^c^	ND	9.04 ± 0.05 ^a^
*β*-resorcylic acid	ND	2.08 ± 0.00 ^b^	2.10 ± 0.06 ^b^	2.79 ± 0.11 ^a^
Chlorogenic acid	ND	ND	ND	0.08 ± 0.01
Ferulic acid	0.40 ± 0.00 ^b^	0.42 ± 0.00 ^a^	ND	ND
Gallic acid	2.85 ± 0.24 ^c^	4.99 ± 0.01 ^b^	2.41 ± 0.07 ^d^	7.84 ± 0.03 ^a^
*p*-coumaric acid	75.13 ± 0.07 ^a^	1.60 ± 0.00 ^c^	1.59 ± 0.00 ^d^	5.60 ± 0.04 ^b^
*p*-hidroxybenzoic acid	0.32 ± 0.01 ^a^	ND	0.33 ± 0.05 ^a^	ND
Protocatechuic acid	ND	ND	3.1 ± 0.02 ^b^	25.12 ± 0.08 ^a^
Rosmarinic acid	0.91 ± 0.02 ^b^	5.49 ± 0.12 ^a^	ND	ND
Sinapic acid	ND	0.95 ± 0.00 ^a^	0.94 ± 0.00 ^b^	ND
Vanillic acid	1.90 ± 0.36	ND	ND	ND
**Flavonoids**				
Apigenin	ND	1.98 ± 0.01	ND	ND
Catechin	1.44 ± 0.11 ^b^	ND	ND	8.84 ± 1.53 ^a^
Phloretin	0.09 ± 0.00 ^a^	0.01 ± 0.00 ^b^	ND	ND
Phloridzin	0.08 ± 0.02 ^a^	ND	ND	0.07 ± 0.00 ^a^
Isorhamnetin	0.97 ± 0.02 ^a^	0.78 ± 0.01 ^b^	ND	ND
Kaempferol	1.65 ± 0.01 ^b^	2.61 ± 0.01 ^a^	ND	ND
Myricetin	ND	1.76 ± 0.00	ND	ND
Naringenin	0.10 ± 0.03 ^a^	0.01 ± 0.01 ^b^	ND	ND
Quercetin	1.94 ± 0.03 ^c^	2.14 ± 0.03 ^a^	8.94 ± 0.04 ^d^	2.02 ± 0.01 ^b^

CAHE: Cardon (*Cylindropuntia rosea*) hydroalcoholic extract; XUHE: Xoconostle ulapa (*Opuntia oligacantha*) hydroalcoholic extract; XCHE: Xoconostle cuaresmeño (*Opuntia matudae*) hydroalcoholic extract; PIHE: Pitaya (*Echinocereus cinerascens*) hydroalcoholic extract; ND = not detected. Values represent the mean ± standard deviation (*n* = 3; *n* refers to analytical replicates). Values with different letters in the same row indicate statistically significant differences at *p* < 0.05, according to the Tukey test.

## Data Availability

The original contributions presented in this study are included in the article/supplementary material. Further inquiries can be directed to the corresponding author(s).

## Supplementary Materials

The following supporting information can be downloaded at https://www.mdpi.com/article/10.3390/metabo15100665/s1: Stastistical_analysisS1: Statistical analysis of the anti-inflammatory effect of four edible cacti flowers from Mexico.
