# Supplementary material for: In Vivo Anti-Inflammatory Activity of Four Edible Cactaceae Flowers from Mexico
Source: Metabolites. 2025 Oct 11;15(10):665. doi: 10.3390/metabo15100665 (PMC12565958; doi:10.3390/metabo15100665)
Supplement: Supplementary file 1 [file metabolites-15-00665-s001.zip › metabolites-3838079-supplementary.pdf]

In vivo anti-inflammatory activity of four edible Cactaceae flowers from Mexico

Christian Alfredo Pensamiento-Niño[1], Alma Delia Hernández-Fuentes[2], Javier Añorve-Morga[3], Arturo Duarte-Sierra[4], Esther Ramírez-Moreno[5], Carolina Guadalupe Sosa-Gutiérrez[6] and Deyanira Ojeda-Ramírez

Inhibition of inflammation exerted by hydroalcoholic extracts of Cacateceae flowers on CD-1 mouse ear edema induced by TPA.

CAHE: cardon (*Cylindropuntia rosea*) hydroalcoholic extract

XUHE: Xoconostle Ulapa variety (*Opuntia oligacantha*) hydroalcoholic extract

XCHE: Xoconostle Cuaresmeño variety (*Opuntia matudae*) hydroalcoholic extract

PIHE: pitaya (*Echinocereus cinerascens*) hydroalcoholic extract

Indo: indomethacin (positive control)

Ctrl: negative control group.

n=4

	% INFLAMATION INHIBITION					
TREATMENT	1	2	3	4	AVERAGE	SD
INDOMETACIN	76.91	83.92	78.41	76.32	78.89	3.47
PIHE	27.32	18.54	12.15	19.13	19.29	6.22
XCHE	38.27	12.99	25.54	19.73	24.13	10.73
XUHE	28.65	30.69	18.89	31.53	27.44	5.83
CAHE	61.2	56.81	66.92	59.87	61.20	4.23
CTRL NEG	0	0	0	0	0.00	0.00

UNNORMALIZED DATA

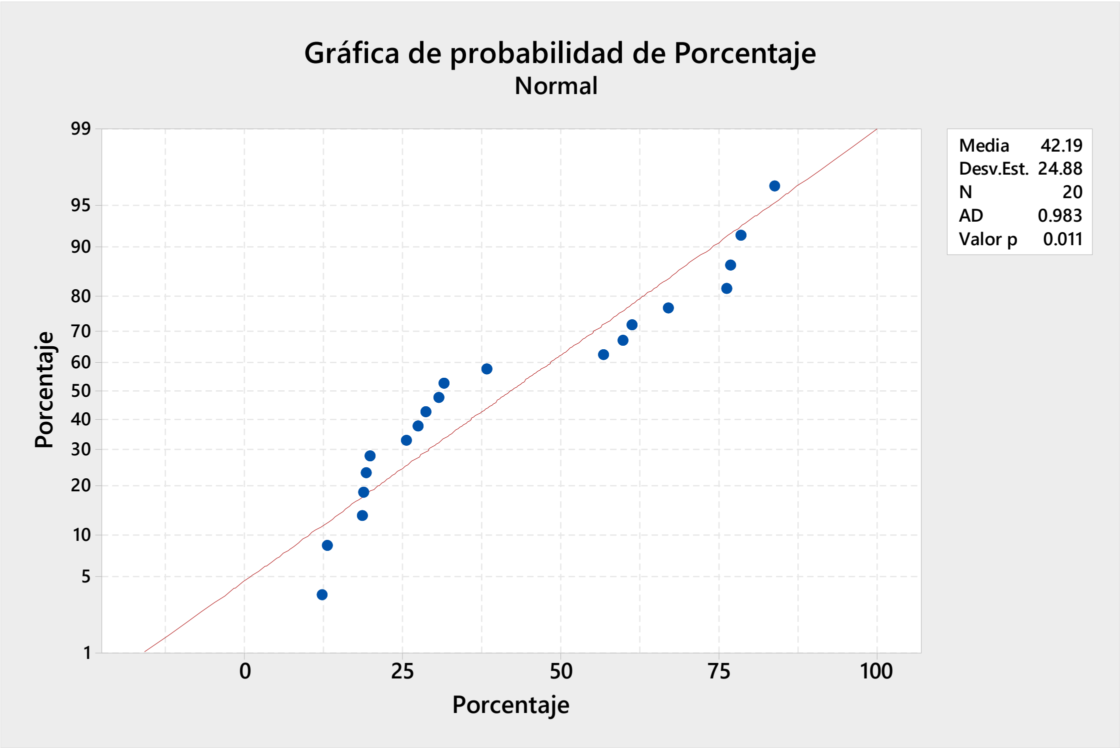

Anderson darling normality test

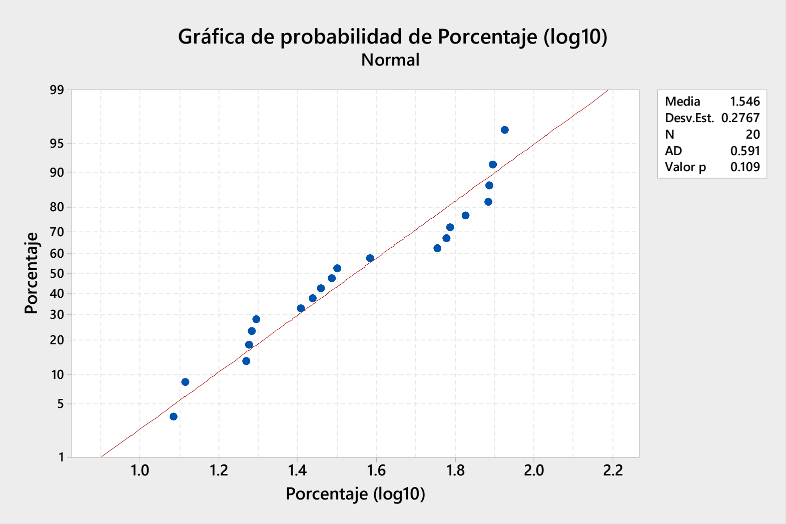

Shapiro wilk normality test

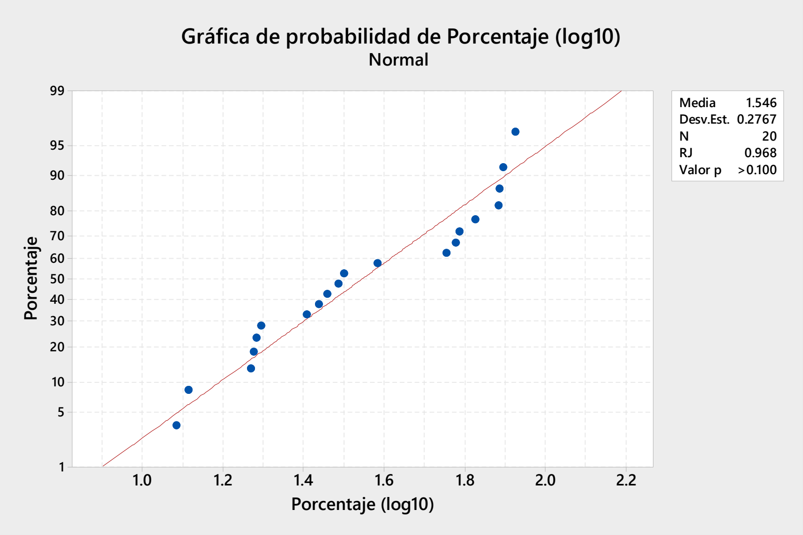

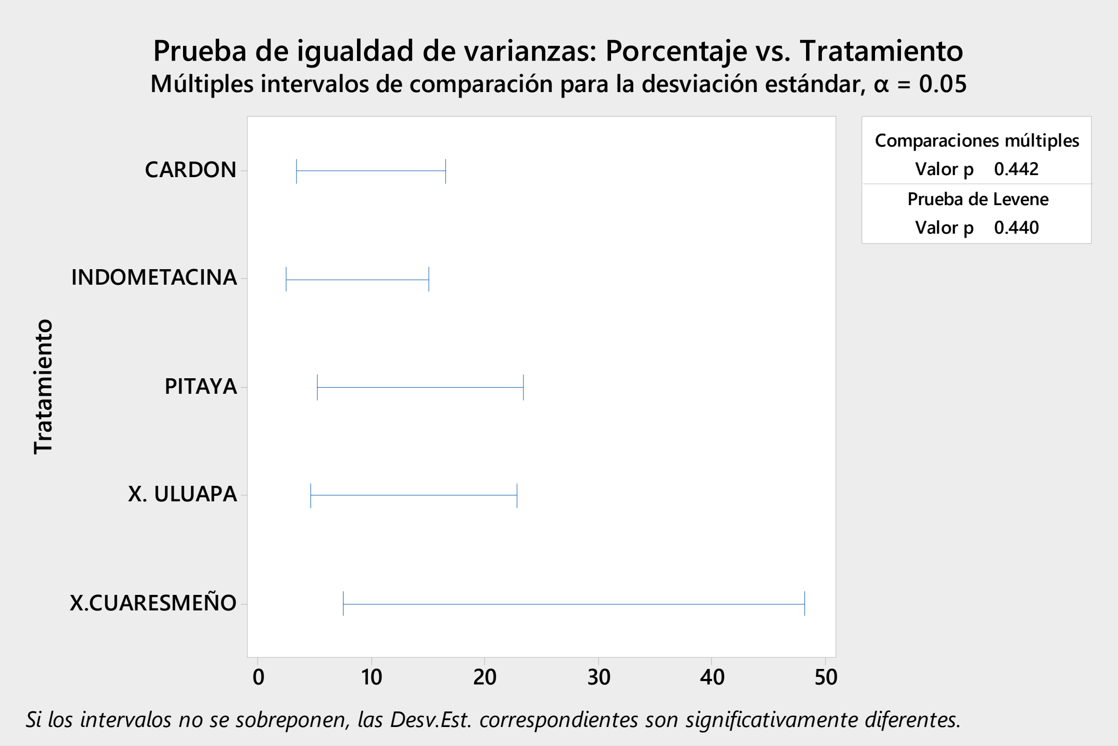

TEST

Prueba de igualdad de varianzas: Porcentaje (log10) vs. Tratamiento

Test for equality of variances: Percentage (log10) vs. Treatment

Método

Hipótesis nula	Todas las varianzas son iguales
Hipótesis alterna	Por lo menos una varianza es diferente
Nivel de significancia	α = 0.05

Intervalos de confianza de Bonferroni de 95% para desviaciones estándar

Method:

Null hypothesis: All variances are equal.

Alternative hypothesis: At least one variance is different.

Significance level: α = 0.05

95% Bonferroni confidence intervals for standard deviations

Tratamiento	N	Desv.Est.	IC
CARDON (CAHE)	4	0.029662	(0.0031859, 0.77566)
INDOMETACINA (INDO)	4	0.018761	(0.0017836, 0.55430)
PITAYA(PIHE)	4	0.144011	(0.0152249, 3.82592)
X. ULUAPA (XUHE)	4	0.103846	(0.0090545, 3.34512)
X.CUARESMEÑO (XCHE)	4	0.196968	(0.0245995, 4.42956)

Nivel de confianza individual = 99%

Pruebas

Método	Estadística de prueba	Valor p
Comparaciones múltiples	—	0.006
Levene	2.07	0.135

NORMALIZED DATA

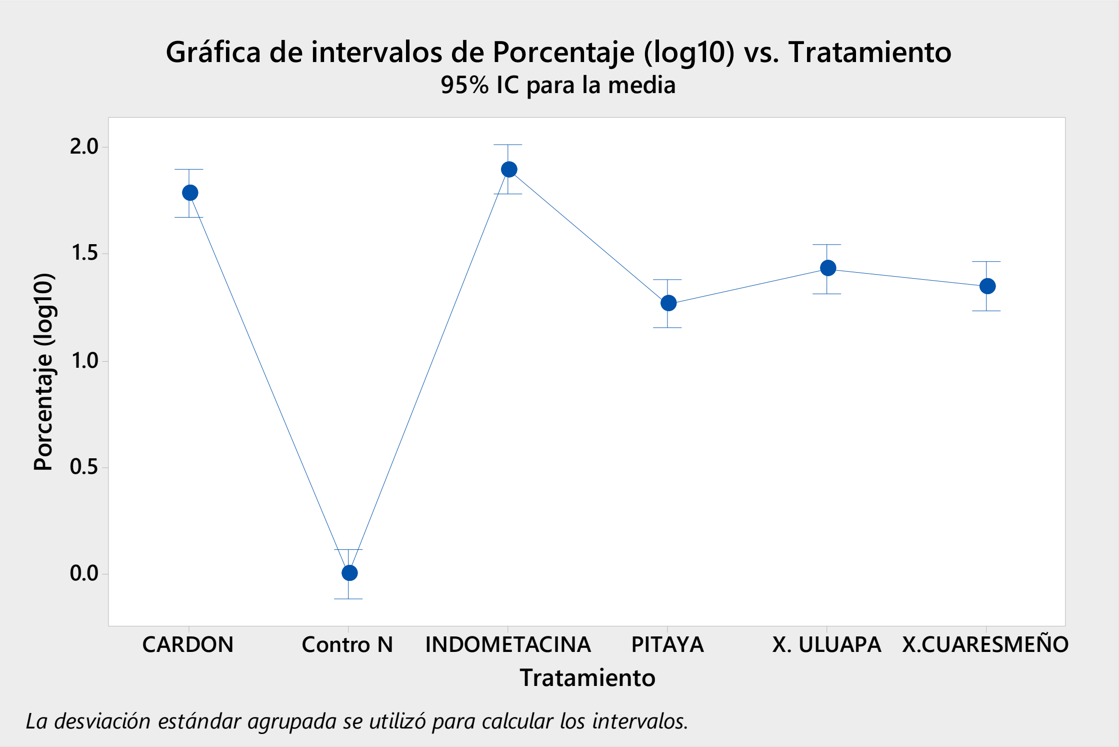

ANOVA de un solo factor: Porcentaje (log10) vs. Tratamiento

Método

Hipótesis nula	Todas las medias son iguales
Hipótesis alterna	No todas las medias son iguales
Nivel de significancia	α = 0.05

Se presupuso igualdad de varianzas para el análisis.

One-Way ANOVA: Percentage (log10) vs. Treatment

Method

Null Hypothesis: All means are equal.

Alternative Hypothesis: Not all means are equal.

Significance Level: α = 0.05

Equal variances were assumed for the analysis.

Información del factor

Factor	Niveles	Valores
Tratamiento	6	CAHE, Control Neg, Indo, PIHE, XUHE, XCHE

Análisis de Varianza

Fuente	GL	SC Ajust.	MC Ajust.	Valor F	Valor p
Tratamiento	5	9.2071	1.84143	154.41	0.000
Error	18	0.2147	0.01193		
Total	23	9.4218			

Resumen del modelo

S	R-cuad.	R-cuad. (ajustado)	R-cuad. (pred)
0.109203	97.72%	97.09%	95.95%

Medias

Tratamiento	N	Media	Desv.Est.	IC de 95%
CARDON (CAHE)	4	1.7860	0.0297	(1.6713, 1.9007)
Contro Neg	4	0.000000	0.000000	(-0.114713, 0.114713)
INDOMETACINA (INDO)	4	1.89671	0.01876	(1.78200, 2.01143)
PITAYA (PIHE)	4	1.2677	0.1440	(1.1530, 1.3824)
X. ULUAPA (XUHE)	4	1.4298	0.1038	(1.3151, 1.5445)
X.CUARESMEÑO (XCHE)	4	1.3497	0.1970	(1.2350, 1.4644)

Desv.Est. agrupada = 0.109203

Comparaciones en parejas de Tukey

Agrupar información utilizando el método de Tukey y una confianza de 95%

Tratamiento	N	Media	Agrupación	
INDOMETACINA (INDO)	4	1.89671	A	
CARDON (CAHE)	4	1.7860	A	
X. ULUAPA (XUHE)	4	1.4298		B

X.CUARESMEÑO (XCHE)	4	1.3497		B	
PITAYA (PIHE)	4	1.2677		B	
Contro Neg	4	0.000000			C

Las medias que no comparten una letra son significativamente diferentes.

Comparaciones en parejas de Fisher

Agrupar información utilizando el método LSD de Fisher y una confianza de 95%

Tratamiento	N	Media	Agrupación		
INDOMETACINA (INDO)	4	1.89671	A		
CARDON (CAHE)	4	1.7860	A		
X. ULUAPA (XUHE)	4	1.4298		B	
X.CUARESMEÑO (XCHE)	4	1.3497		B	
PITAYA (PIHE)	4	1.2677		B	
Control Neg	4	0.000000			C

Las medias que no comparten una letra son significativamente diferentes.

Datos sin normalizar

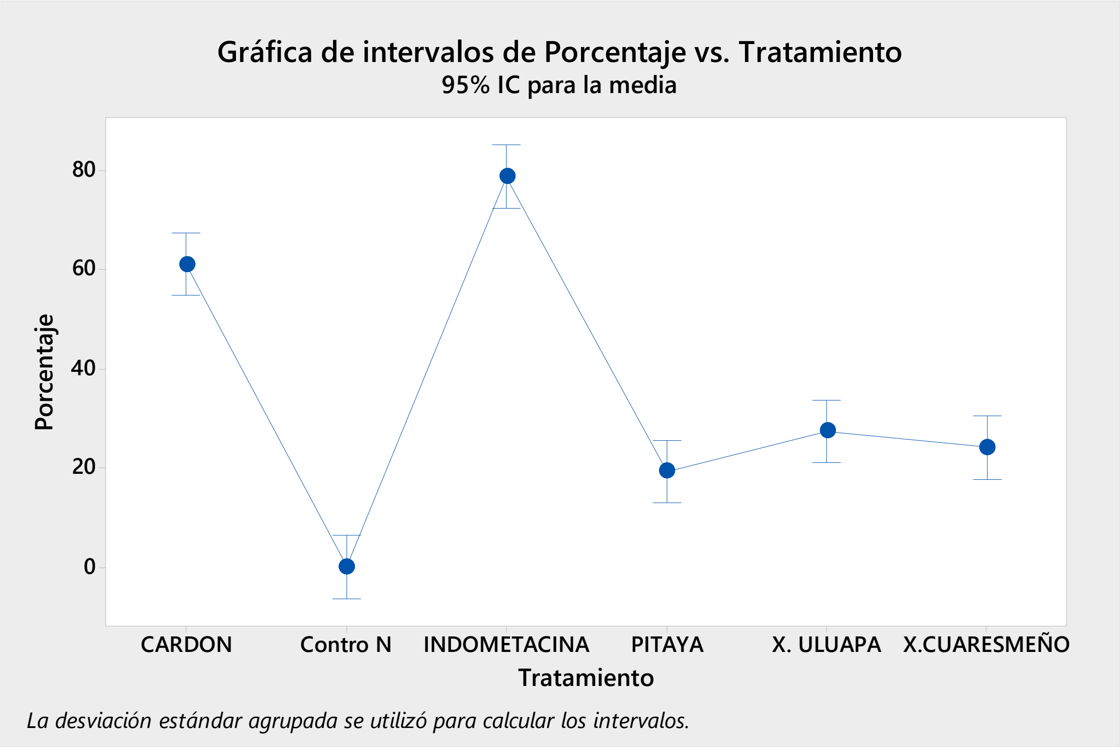

ANOVA de un solo factor: Porcentaje vs. Tratamiento

Método

Hipótesis nula	Todas las medias son iguales
Hipótesis alterna	No todas las medias son iguales
Nivel de significancia	α = 0.05

Se presupuso igualdad de varianzas para el análisis.

Información del factor

Factor	Niveles	Valores
Tratamiento	6	CARDON, Contro N, INDOMETACINA, PITAYA, X. ULUAPA, X.CUARESMEÑO

Análisis de Varianza

Fuente	GL	SC Ajust.	MC Ajust.	Valor F	Valor p
Tratamiento	5	17039.4	3407.87	93.92	0.000
Error	18	653.1	36.28		
Total	23	17692.5			

Resumen del modelo

S	R-cuad.	R-cuad. (ajustado)	R-cuad. (pred)
6.02361	96.31%	95.28%	93.44%

Medias

Tratamiento	N	Media	Desv.Est.	IC de 95%
CARDON	4	61.20	4.23	(54.87, 67.53)
Contro N	4	0.000000	0.000000	(-6.327564, 6.327564)
INDOMETACINA	4	78.89	3.47	(72.56, 85.22)
PITAYA	4	19.29	6.22	(12.96, 25.61)
X. ULUAPA	4	27.44	5.83	(21.11, 33.77)
X.CUARESMEÑO	4	24.13	10.73	(17.80, 30.46)

Desv.Est. agrupada = *6.02361*

Comparaciones en parejas de Tukey

Agrupar información utilizando el método de Tukey y una confianza de 95%

Tratamiento	N	Media	Agrupación	
INDOMETACINA	4	78.89	A	
CARDON	4	61.20		B

X. ULUAPA	4	27.44	C
X.CUARESMEÑO	4	24.13	C
PITAYA	4	19.29	C
Contro N	4	0.000000	D

Las medias que no comparten una letra son significativamente diferentes.

Comparaciones en parejas de Fisher

Agrupar información utilizando el método LSD de Fisher y una confianza de 95%

Tratamiento	N	Media	Agrupación
INDOMETACINA	4	78.89	A
CARDON	4	61.20	B
X. ULUAPA	4	27.44	C
X.CUARESMEÑO	4	24.13	C
PITAYA	4	19.29	C
Contro N	4	0.000000	D

Las medias que no comparten una letra son significativamente diferentes.

Comparaciones múltiples de Dunnet con un control

Agrupar información utilizando el método de Dunnett y una confianza de 95%

Tratamiento	N	Media	Agrupación
Contro N (control)	4	0.000000	A
INDOMETACINA	4	1.89671	
CARDON	4	1.7860	
X. ULUAPA	4	1.4298	
X.CUARESMEÑO	4	1.3497	
PITAYA	4	1.2677	

Las medias no etiquetadas con la letra A son significativamente diferentes de la media del nivel de control.

Comparaciones múltiples de Dunnet con un control

Agrupar información utilizando el método de Dunnett y una confianza de 95%

Tratamiento	N	Media	Agrupación
INDOMETACINA (control)	4	1.89671	A
CARDON	4	1.7860	A
X. ULUAPA	4	1.4298	

X.CUARESMEÑO	4	1.3497
PITAYA	4	1.2677
Control N	4	0.000000

Las medias no etiquetadas con la letra A son significativamente diferentes de la media del nivel de control.
